# Glycogen Synthase Kinase 3β and Activin/Nodal Inhibition in Human Embryonic Stem Cells Induces a Pre-Neuroepithelial State That Is Required for Specification to a Floor Plate Cell Lineage

**DOI:** 10.1002/stem.1204

**Published:** 2012-08-21

**Authors:** Mark Denham, Chris Bye, Jessie Leung, Brock J Conley, Lachlan H Thompson, Mirella Dottori

**Affiliations:** a Centre for Neuroscience Research, Department of Anatomy and Neuroscience, University of MelbourneParkville, Australia; b Florey Neuroscience InstitutesParkville, Australia

**Keywords:** Floor plate, FOXA2, Human embryonic stem cells, Sonic hedgehog, GLI

## Abstract

The floor plate is one of the major organizers of the developing nervous system through its secretion of sonic hedgehog (Shh). Although the floor plate is located within the neural tube, the derivation of the floor plate during development is still debatable and some studies suggest that floor plate cells are specified by Shh in a temporarily restricted window different to neuroepithelial cells. Using human embryonic stem cells (hESC) as a model of neurogenesis, we sought to determine how floor plate cells may be temporarily specified by SHH signaling during human embryogenesis. We found that inhibition of both GSK3β and activin/nodal pathways in hESC induces a cellular state of SOX2+/PAX6− expression, we describe as “pre-neuroepithelial.” Exposure of SHH during this pre-neuroepithelial period causes the expression of GLI transcription factors to function as activators and consequently upregulate expression of the floor plate marker, FOXA2, while also supressing PAX6 expression to inhibit neuroepithelial fate. FOXA2+ cells were able to efficiently generate mesencephalic dopaminergic neurons, a floor plate derivative. Overall, this study demonstrates a highly efficient system for generating floor plate cells from hESC and, most importantly, reveals that specification of floor plate cells is temporally dependent, whereby it occurs prior to the onset of PAX6 expression, within a pre-neuroepithelial stage. Stem Cells*2012;30:2400–2411*

## INTRODUCTION

The floor plate is a secondary organizer that secretes Sonic hedgehog (Shh) in order to pattern ventral regions of the developing neural tube [1]. Cells within the floor plate express the forkhead winged helix transcription factor FoxA2 and are predominantly non-neurogenic except within the midbrain regions of the neural tube where they give rise to mesencephalic dopaminergic neurons [2]. The generation of the floor plate itself is controversial and also seems to be species dependent [3–5]. The long-standing model of ventralization of the neural tube has been that, during gastrulation, neuroepithelial cells exposed to increasing concentrations of Shh give rise to more ventral neural progenitor subtypes [5]. However, certain studies in chick and mouse embryos have indicated that this model does not hold true for specification of the floor plate, and that high concentrations of Shh signaling may not in itself be sufficient to induce specification of floor plate cells in the neural tube [3, 6]. Studies in chick embryos have demonstrated that exposing the dorsal neural tube to Shh signaling promotes ventralization to motor neurons but not to a floor plate fate [6], suggesting that high levels of Shh itself are not enough to confer commitment to a floor plate identity. Other studies contradict this theory by showing that Shh can promote a floor plate phenotype in explant cultures from embryonic rat brain [7]. Shh, however, is critical for floor plate specification as Shh knockout mice do not develop a floor plate and have severe dorsoventral patterning defects [8]. More recently it was demonstrated in chick that in addition to requiring high levels of Shh concentration, the timing and duration of Shh signaling is critical for specifying cells toward a floor plate fate [9]. These studies suggest that floor plate cells are specified by Shh in a temporarily restricted window, earlier than the neighboring neuroepithelial cells. The critical timing of SHH signaling was also reported by Fasano et al. [10] using human embryonic stem cells (hESC). In this study, they demonstrated that early exposure of SHH promoted floor plate fate at the expense of differentiation toward anterior neuroectoderm, and that delaying SHH treatment to cultures from day 1 to day 3 resulted in a significant reduction in FOXA2+ cells [10].

If the timing of Shh exposure is critical for floor plate specification, then this also questions what are the parent progenitors that generate floor plate cells. Elegant studies in the chick embryo describe a “dual origin” of floor plate cells, derived from both Hensen's node as well as neuroectoderm (Sox1+ cells), defined as medial and lateral floor plate cells, respectively [3]. Interestingly, the medial floor plate cells can give rise to lateral floor plate cells but not vice versa. Subsequent studies in the chick further defined the origins of anterior floor plate cells and determined that they were derived during gastrulation, from the prenodal epiblast region that lies anterior to Hensen's node [11]. The timing and origin of floor plate cells in mammalian development, particularly in human embryos, remains to be determined. Also, differences in gene expression profiles across species of early pan-neuroepithelial markers, such as Pax6 and Sox1, further compounds this investigation. In human embryos, PAX6 is expressed in the neural plate, which is the earliest neural structure to form [12]. PAX6 is also expressed in early neuroepithelial cells derived from hESC, and forced expression of PAX6 in hESC drives their fate to neural [12–14]. In contrast, Sox1 is expressed prior to Pax6 in the neural plate of mice embryos [15]. Furthermore, forced Pax6 expression in mouse ESCs does not induce their neural fate [12, 16]. Since the floor plate develops at a similar developmental stage as the neural plate, there may be fundamental differences between humans and other species in the molecular pathways involved in generating floor plate cells.

Our studies provide evidence for the existence of a pre-neuroepithelial cellular state, that is, prior to PAX6 expression, whereby SHH signaling mediates specification to a floor plate fate in the human system. Most significantly, we identified the key signaling pathways activated in hESC to attain this pre-neuroepithelial state. Small molecule inhibition of both glycogen synthase kinase 3β (GSK3β) and Activin/Nodal signaling is able to efficiently direct hESC to nonpluripotent (OCT4−/NANOG−), pre-neuroepithelial (SOX2+/PAX6−) progenitors. We show that these pre-neuroepithelial cells can upregulate expression of GLI1 and specify their fate to floor plate in response to high levels of SHH signaling. Moreover, continued SHH signaling is not required for commitment to a floor plate state and that instead a state of cellular memory is induced in the progenitor cells through their exposure to SHH signaling. These studies reveal the events that delineate cell lineage commitment and specification toward floor plate versus neuroepithelium.

## MATERIALS AND METHODS

### hESC Culture

HES-3 (WiCell, Madison, WI, http://www.wicell.org), ENVY-HES-3 (BioTime, ES Cell International), and H9 (WA-09, WiCell) cell lines were cultured as previously described [17, 18]. Briefly, hESCs were cultured on mitomycin-C-treated mouse embryonic fibroblasts (MEFs) in hESC medium consisting of high-glucose Dulbecco's modified Eagle's medium (DMEM) without sodium pyruvate, supplemented with insulin/transferrin/selenium 1%, β-mercaptoethanol 0.1 mM, nonessential amino acids (NEAA) 1%, glutamine 2 mM, penicillin 25 U/ml, streptomycin 25 μg/ml (all from Invitrogen, Carlsbad, CA, http://www.invitrogen.com), and fetal calf serum (FCS) 20% (Hyclone, Logan, UT, http://www.hyclone.com) or on mitomycin-C treated human foreskin fibroblasts (HFF) in knockout serum replacer (KSR) media consisting of DMEM/nutrient mixture F-12, supplemented with β-mercaptoethanol 0.1 mM, NEAA 1%, glutamine 2 mM, penicillin 25 U/ml, streptomycin 25 μg/ml, and KSR 20% (all from Invitrogen). All cells were cultured at 37°C 5% CO_2_. Colonies were mechanically dissected every 7 days and transferred to freshly prepared MEFs or HFFs. Media was changed every second day.

### Neural Induction in Defined Medium

hESCs were mechanically dissected into pieces approximately 0.5 mm in diameter and transferred to laminin-coated organ culture plates in N2B27 medium containing 1:1 mix of neurobasal medium with DMEM/F12 medium, supplemented with insulin/transferrin/selenium 1%, N2 1%, retinol-free B27 1%, glucose 0.3%, penicillin 25 U/ml, and streptomycin 25 μg/ml (all from Invitrogen) for 11 days [19]. SB431542 (10 μM, Tocris) was added to the media only for the first 4 days followed by the addition of basic fibroblast growth factor (20 ng/ml, R&D, Minneapolis, MN, http://www.rndsystems.com) for the remaining 7 days. For conditions C–H, cultures were grown on laminin for the first 4 days after which they were dissected into 0.5 mm pieces and cultured in suspension in low-attachment 96-well plates (Corning, Acton, MA, http://www.corning.com/lifesciences) in N2B27 medium. Where indicated, cultures were supplemented with either: smoothened agonist (SAG; 400 nM, Merck, Whitehouse Station, NY, http://www.merck.com), GSK3β inhibitor CHIR99021 (3 μM, Stemgent), FGF8 (100 ng/ml, R&D), or MEK1/2 inhibitor PD0325901 (1 μM, Tocris). For 21- and 42-day neuronal differentiation, the last 10 and 14 days, respectively, of culture, spheres were plated onto laminin-coated dishes in N2B27 medium without factors. Schematics of conditions are shown in relevant figures and further described in Supporting Information Methods.

### Fluorescent Activated Cell Sorting Analysis

hESCs or differentiated derivatives were dissociated into single cells with TrypLE Express (Invitrogen), centrifuged and resuspended in 4% paraformaldehyde (PFA) for 10 minutes, and subsequently washed in phosphate buffered saline (PBS), and permeabilized with 0.25% Triton X in PBS (PBT). The following primary antibodies were used: mouse anti-Nkx2.1 (1:20, Abcam, Cambridge, U.K., http://www.abcam.com), goat anti-FoxA2 (1:10, Santa Cruz Biotechnology, Santa Cruz, CA, http://www.scbt.com), goat anti-Sox2 (1:30, R&D), mouse anti-Oct4 (1:20, Santa Cruz Biotechnology). Antibodies were diluted in blocking solution (PBT with 10% FCS), and cells were centrifuged and resuspended in primary antibody solution overnight at 4°C. Following three 10-minute washes in PBT, cell were resuspended in the corresponding secondary antibodies for 30 minutes at room temperature (RT): donkey anti-goat Cy5, donkey anti-mouse Cy2 (1:400, Jackson ImmunoResearch, West Grove, PA, http://www.jacksonimmuno.com), followed by a wash in blocking solution before being immediately sorted using an LSR Fortessa cell analyzer.

### Immunostaining

Cell monolayers and neurospheres were fixed in 4% PFA for 20 minutes at 4°C and then washed briefly in PBS. Neurospheres or day 4 cell aggregates were embedded in Tissue-Tek OCT compound (Labtek), cut at 10 μm on a cryostat, and sections were placed on superfrost slides. Sections or culture dishes were blocked for 1 hour at RT in blocking solution. The following primary antibodies were used: goat anti-FoxA2 (1:300, Santa Cruz Biotechnology), mouse anti-Nkx2.1 (1:300, Abcam) goat anti-Sox2 (1:500, R&D), rabbit anti-Otx2 (1:4,000, Merck Millipore), rabbit anti-TH (1:1,000, Pel Freez), mouse anti-Tuj1 (1:500, Promega, Madison, WI, http://www.promega.com), mouse anti-Oct4 (1:100 Santa Cruz Biotechnology), mouse anti-Nanog (1:50, eBiosciences), mouse anti-Tra-1-81 (1:75, Merck Millipore), mouse anti-Tra-1-60 (1:75, Merck Millipore), rabbit anti-β-catenin (1:100, Cell Signaling Technology, Beverly, MA, http://www.cellsignal.com), rabbit anti-FOXG1 (1:50, Abcam), mouse anti-Pax6 (1:40), mouse anti-En1 (1:40), and mouse anti-Nkx6.1 (1:10, all from DSHB). Antibodies were diluted in blocking solution incubated on sections overnight at 4°C. Following three 10-minute washes in PBT, the corresponding secondary antibodies were applied for 1 hour at RT: anti-goat Cy3, anti-goat Cy5, anti-mouse Cy2, anti-mouse Cy3, anti-mouse Cy5 anti-rabbit Cy3, and anti-rabbit Cy5 (1:400, Jackson ImmunoResearch). Sections and cultures were counterstained for 5 minutes with 4′,6-diamidino-2-phenylindole (DAPI) (1 μg/ml, Sigma). Slides were mounted in PVA-DABCO (2.5% DABCO, 10% polyvinylalcohol (Sigma; Type II), 5% glycerol, 25 mM Tris buffer, pH 8.7) for viewing under an immunofluorescent microscope (Olympus), and images were captured using the Cell-M software. Confocal microscopy was performed using an Olympus FV1000 Confocal Microscope. The image was then reconstructed as an intensity projection over the Z-axis using Olympus FV10-ASW 2.0 Viewer software.

### Real-Time Polymerase Chain Reaction

hESC, day 4 and day 11 time points of the various differentiation conditions were collected by mechanical isolation, all conditions were analyzed from three independent biological replicates. Total RNA was isolated from cells using the RNeasy Mini RNA Extraction Kit (Qiagen, Hilden, Germany, http://www1.qiagen.com) and reverse transcribed using the SuperScript III quantitative reverse transcription polymerase chain reaction (qRT-PCR) Kit (Invitrogen). Real-time PCR was carried out using the SYBR GreenER qPCR SuperMix Universal (Invitrogen) on a Rotor-Gene 6000 (Corbett Life Science) and analyzed using the ΔΔCT method [20]. Fold changes were expressed relative to the hESC group. Primers for the following genes were used: FOXA2, SHH, GLI1, GLI2, GLI3, BRACHYURY, SOX17 and the housekeeping gene HPRT1. The primer sequences are available as Supporting Information data.

### Statistical Analysis

Fluorescent activated cell sorting (FACS) analysis was performed on at least 10,000 events per replicate. These events were counted after gating out cell debris and doublets on the forward and side scatter. For QPCR and FACS analysis, experiments were repeated at least three times. One-way ANOVAs were performed for statistical analyses.

## RESULTS

### Standard Neural Induction Using Activin/Nodal Inhibition and SHH Agonist Is not Sufficient for Floor Plate Specification

**Figure 1:**
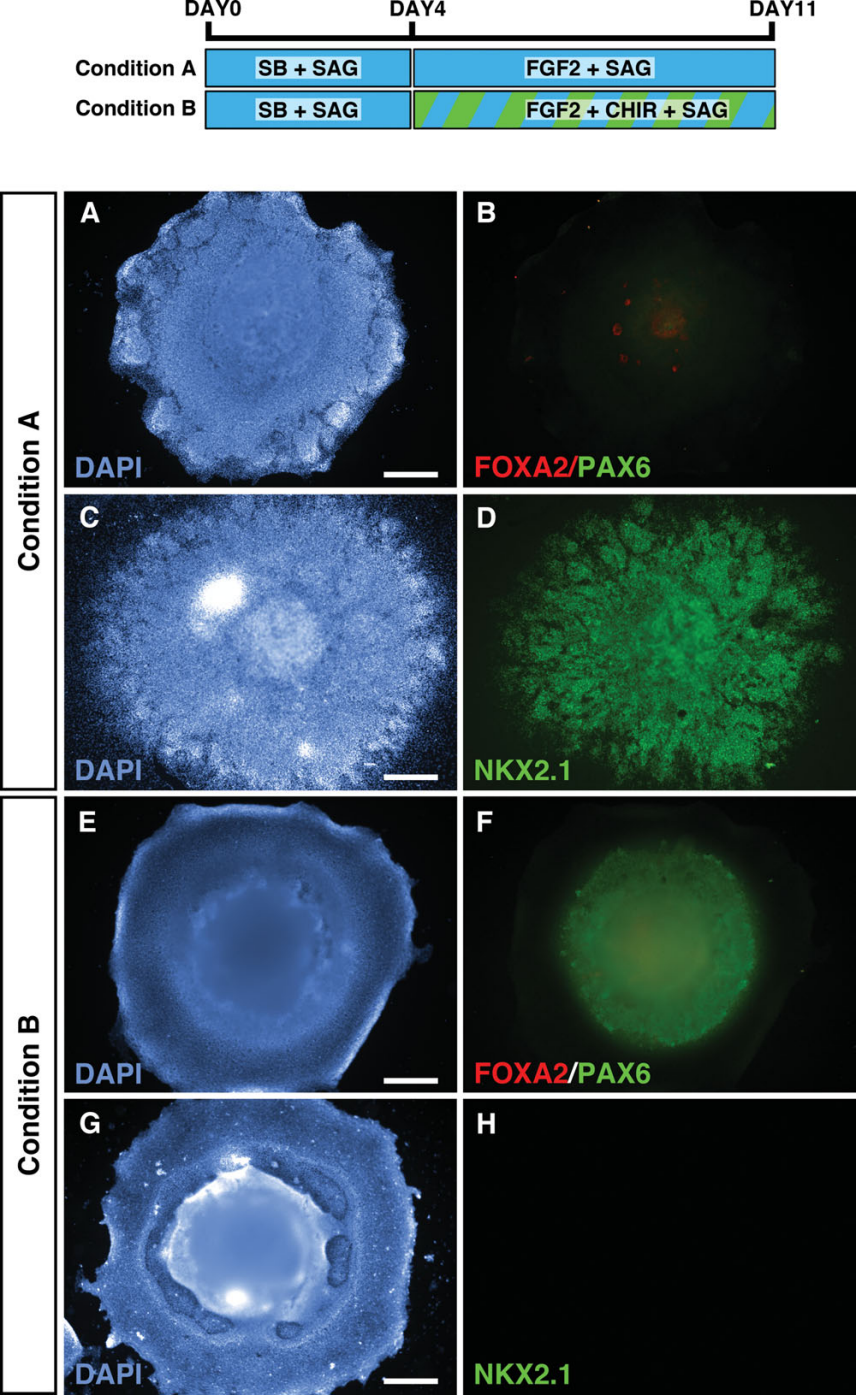
Early treatment with sonic hedgehog (SHH) agonist is not sufficient for floor plate specification. **(A–D):** Neural induction in defined media with SAG yields few FOXA2+ cells (condition A). **(E–H):** In condition B, addition of the glycogen synthase kinase 3β inhibitor CHIR from days 4 to 11 resulted in no detectable NKX2.1+ cells and expansion of the neuroepithelial marker PAX6. No detectable FOXA2+ cells were found. Scale bars = 500 μm. Abbreviations: DAPI, 4′,6-diamidino-2-phenylindole; SAG, smoothened agonist; SB, SB431542.

Standard protocols for hESC neural induction involves the use of a coculture system using stromal cells as a feeder or, alternatively, in feeder-free conditions when hESC are treated with the Activin/Nodal inhibitor SB431542 (which blocks phosphorylation of the ALK4, ALK5, and ALK7 receptors) and/or Noggin (which blocks signaling through the bone morphogenetic protein (BMP) receptors) [19, 21, 22]. We, and others, have previously reported that supplementation with recombinant SHH is required to induce hESC neural differentiation toward ventral cell types [22, 23]. However, these studies show that recombinant SHH is not efficient at inducing floor plate cells from hESC, even when administered at high concentrations. One possible explanation is that the SHH recombinant protein is not lipid modified and thus cannot transduce a signal equivalent to endogenous levels [24, 25]. This may be circumvented with the use of a small molecule agonist of the SHH pathway, SAG, which directly binds to the smoothened receptor and thus activates a SHH responsive signal [26]. To assess the effectiveness of SAG in inducing floor plate cells from hESC, the laminin substrate neural induction system was used, as previously described [22]. Briefly, this induction system involves plating hESC on a feeder-free, laminin substrate in neural media for 11 days, where the first 4 days includes treatment with the small molecule Activin/Nodal inhibitor, SB431542, to promote neural induction. Using this system, SAG treatment was included throughout the 11-day protocol (Fig. 1, condition A). It was found that after 11 days of SAG treatment, 75.18% ± 7.9% SEM of the colonies expressed the ventral marker, NKX2.1, with a corresponding loss of the early neuroepithelial marker, PAX6 [12] (Fig. 1A–1D; Supporting Information Fig. 1). However, despite this ventralization by SAG treatment, almost no FOXA2+ cells were observed (Fig. 1B), suggesting that activation/repression of alternative signaling pathways may be needed to efficiently induce floor plate specification under these conditions.

### GSK3β Inhibitor Prevents the Onset of PAX6 During Neural Induction

Our previous studies showed that intrinsic ectopic expression of GLI1 was able to induce floor plate cells in hESC-derived neuroepithelial cells by directly activating the SHH signaling cascade [22]. Although Q-PCR analyses of SAG treatment (condition A) at day 4 shows a significant increase in GLI1, there was also a corresponding significant increase in GLI2 and GLI3 factors (*p* < .05, Supporting Information Fig. 2). One possibility for the lack of FOXA2 expression in the SAG-treated cultures is that the repressive activity of GLI2 and GLI3 may be overriding the GLI1 pathway. Suppressor of fused (Sufu) is an essential negative regulator of the hedgehog pathway and is involved in the processing of Gli2/3 into their transcriptional repressors [27–29]. Phosphorylation of Sufu is required for its stabilization and is mediated by protein kinase A (PKA) and GSK3β, with GSK3β also able to form a trimolecular complex with Gli3 and Sufu for the processing of Gli3 into repressors [30, 31]. Thus, an alternate approach may be to prevent the repressive activity of the hedgehog pathway in parallel to direct activation with SAG.

**Figure 2:**
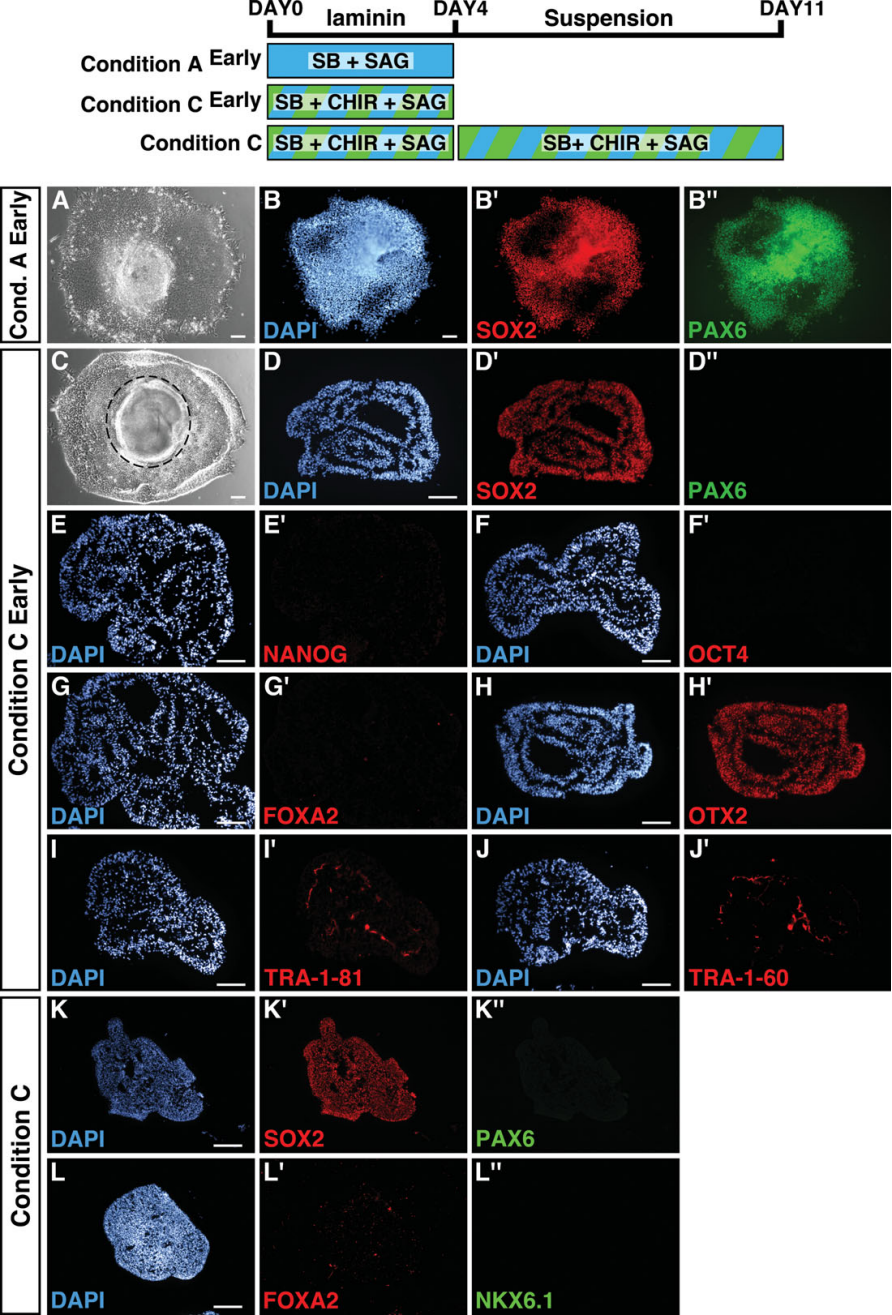
GSK3β and activin/nodal inhibition results in a pre-neuroepithelial state. **(A–B″):** In condition A^Early^, at day 4, cells coexpress neuroepithelial markers, SOX2 and PAX6. **(C):** Cultures treated with SB431542, CHIR, and SAG (condition C^Early^) results in a cellular aggregate arising in the central regions of the colony by day 4 (dashed line). **(D–J′):** Aggregates were harvested at day 4 and examined by immunofluorescence for SOX2 (D′), PAX6 (D″), NANOG (E′), OCT4 (F′), FOXA2 (G′), OTX2 (H′), TRA-181 (I′), and TRA-1-60 (J′). **(K–L″):** Aggregates obtained from SB, CHIR, and SAG-treated cultures were harvested at day 4 and cultured in suspension to day 11 (condition C). At day 11, cells were stained for SOX2 (K′), PAX6 (K″), FOXA2 (L′), and NKX6.1 (L″). Scale bars = 100 μm. Abbreviations: CHIR, CHIR-99021; DAPI, 4′,6-diamidino-2-phenylindole; SAG, smoothened agonist; SB, SB431542.

The small molecule CHIR-99021 (referred to as “CHIR”) is a potent inhibitor of GSK3β and GSK3α. We propose that blocking the action of GSK3β by CHIR may prevent stabilization of SUFU and inhibit its ability to effectively process the GLI2/3 into their transcriptional repressive states, thus shifting the balance toward a GLI activation state and hence allowing greater efficiency of SHH signaling through SAG. To test this model, CHIR was added at day 4 during hESC neural induction and remained supplemented in the culture until day 11 (Fig. 1, condition B). Day 4 was chosen as a time point for administering CHIR based on previous studies suggesting that CHIR treatment may help maintain hESC pluripotency under certain conditions [32]. At day 11, colonies were analyzed for expression of ventral neural markers, including FOXA2. It was found that, unlike condition A, there was no increase in the ventral marker NKX2.1 but instead the expression of the early neuroepithelial marker PAX6 was observed, and very few FOXA2+ cells were observed (Fig. 1E–1H).

One possibility for the lack of FOXA2 expression in the CHIR-treated cultures described above is the timing of CHIR exposure. It may be that commitment toward a neuroepithelial lineage has already occurred by day 4 and thus precluding subsequent floor plate specification. To investigate this hypothesis, our neural induction protocol (condition A) was analyzed at day 4 for expression of early neuroepithelial markers, PAX6 and SOX2. Rapid induction of PAX6 and SOX2 was found at 4 days with the SB431542 inhibition together with SAG, suggesting an early commitment toward a neuroepithelial lineage (Fig. 2A–B″). Given these results, the neural induction protocol was modified to include CHIR treatment from day 0 (condition C). After 4 days of combined SAG, SB431542, and CHIR treatment, cells within the central regions of the colony differentiated into aggregate structures, which could be mechanically harvested (Fig. 2C). Immunostaining analyses of these cell aggregates showed downregulation of the pluripotent markers, OCT4, NANOG, TRA-1-60 and TRA-1-81, however, expression of the pluripotent/neural marker, SOX2, was maintained (SOX2+ cells were 89.17% ± 2.7% SEM and OCT4+ cells were 0.91% ± 0.12% SEM, Fig. 2C–2J′; Supporting Information Fig. 3). Cells surrounding the aggregate were also OCT4−/SOX2+ (data not shown). Interestingly, OTX2 was expressed in the cellular aggregates (an epiblast and anterior neural patterning marker) although no PAX6+ cells were observed (Fig. 2H′, 2D″). Despite an absence of PAX6 expression, no FOXA2+ cells were found at this stage in these conditions (Fig. 2G′).

**Figure 3:**
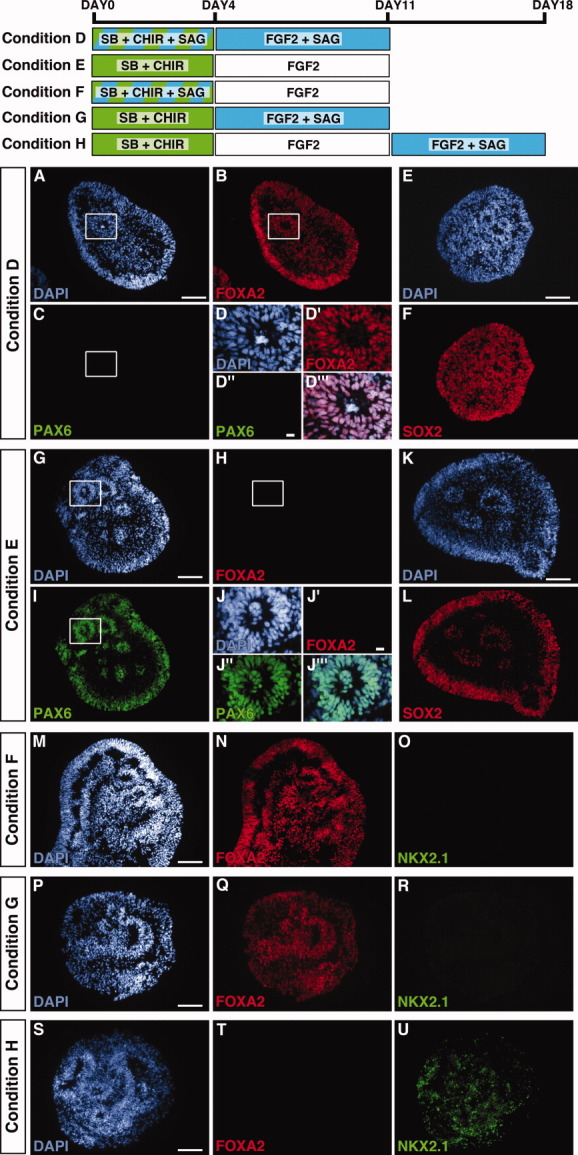
A pre-neuroepithelial stage can be differentiated toward floor plate or neuroepithelial fates. **(A–F):** Condition D cells were examined by immunofluorescence for FOXA2 (B) and PAX6 (C) expression, DAPI (A), box insert (D–D″′). Condition D was also examined for SOX2 expression (F), DAPI (E). **(G–L):** Condition E cells were examined by immunofluorescence for FOXA2 (H) and PAX6 (I) expression, DAPI (G), box insert (J–J″′). Condition E was also examined for SOX2 expression (L), DAPI (K). **(M–O):** Condition F was examined by immunofluorescence for FOXA2 (N) and NKX2.1 (O) expression, DAPI (M). Condition G was examined by immunofluorescence for FOXA2 **(Q)** and NKX2.1 **(R)** expression, DAPI **(P)**. Condition H was examined by immunofluorescence for FOXA2 **(T)** and NKX2.1 **(U)** expression, DAPI **(S)**. Condition D, F, and G all result in high proportions of FOXA2+ cells. Condition E results in PAX6+ cells, and condition H results in NKX2.1+ cells. Scale bars: A–C, E–I, K–U = 100 μm and D–D″′, J–J″′ = 10 μm. Abbreviations: CHIR, CHIR-99021; DAPI, 4′,6-diamidino-2-phenylindole; SAG, smoothened agonist; SB, SB431542.

To determine whether longer exposure of CHIR is needed to induce FOXA2 expression, hESC cultures were treated with SAG and CHIR from day 0 and maintained in these conditions for an additional 11 days (Fig. 2, condition C). Cultures analyzed at day 11 still did not show any significant numbers of FOXA2+ cells (Fig. 2L′). Interestingly, progenitors appeared to remain in a similar state as examined at day 4, that is, SOX2+/PAX6− cells (Fig. 2K–K″). Taken together, these data suggest that early inhibition of the GSK3β pathway during neural induction in hESC can block differentiation toward neuroepithelial cell types and maintain the cells in a state we define as being “pre-neuroepithelial.”

### Transient and Early Exposure of CHIR Results in Highly Efficient Generation of FOXA2+ Cells

**Figure 4:**
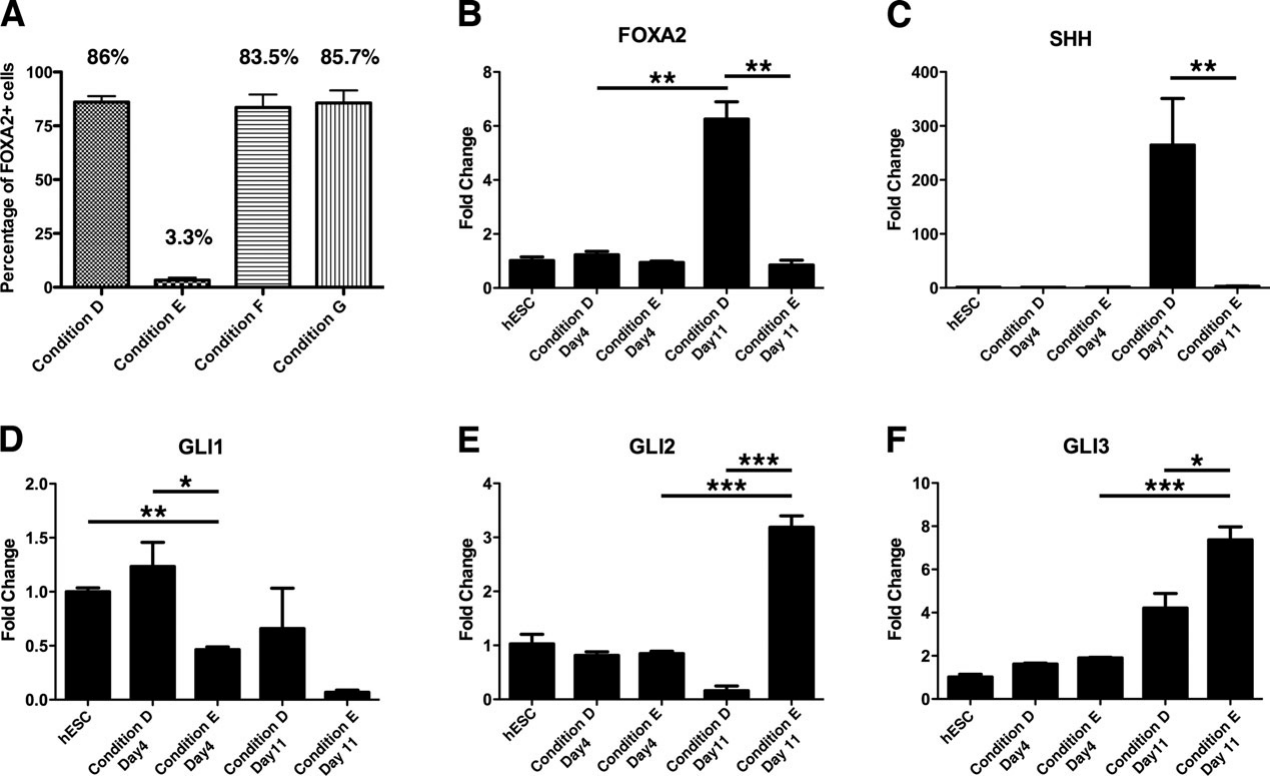
Fluorescent activated cell sorting (FACS) quantification of conditions D–G for FOXA2 at day 11 and quantitative polymerase chain reaction (qPCR) analysis of conditions D and E at day 4 and day 11 relative to hESC. **(A):** FACS analysis of conditions D, F, and G all yielded high percentages of FOXA2+ cells, 86% (±2.8 SEM), 83.5% (±5.9 SEM), and 85.7% (±5.7 SEM), respectively. Condition E resulted in few (3.3%; ±1.2 SEM) FOXA2+ cells, significantly lower than the other conditions (*p* < .0001). **(B–F):** Q-PCR analysis of conditions D and E at day 4 and 11. Q-PCR for FOXA2 (B), SHH (C), GLI1 (D), GLI2 (E), and GLI3 (F). Significant differences of relevant comparisons are indicated: *, **, *** indicated *p* values of <.05, < .005 and <.0005, respectively. Not all significant differences are indicated. Abbreviation: hESC, human embryonic stem cell.

The long-term exposure of CHIR during neural induction may not allow further differentiation of SOX2+/PAX6− progenitors to a floor plate fate. To test this hypothesis, CHIR treatment was applied to the neural induction cultures for the first 4 days only, together with SAG, after which CHIR was removed. The cultures were then further treated with SAG and FGF only for an additional 7 days (Fig. 3, condition D). Immunostaining analyses at the day 11 time point of these conditions showed 86% ± 2.8% SEM FOXA2+ cells (Figs. 3B, 3D′, 4A; Supporting Information Fig. 4). The cells were also negative for PAX6 expression (Fig. 3C, 3D″). Thus, early transient exposure of CHIR results in PAX6− progenitors, which can be efficiently ventralized into FOXA2+ cells through activation of the SHH pathway. In addition, at day 11 the FOXA2+ cultures showed no significant expression of the endoderm and mesoderm markers, SOX17 and BRACHYURY, respectively, confirming that they are of the floor plate progenitor lineage (Supporting Information Fig. 5).

**Figure 5:**
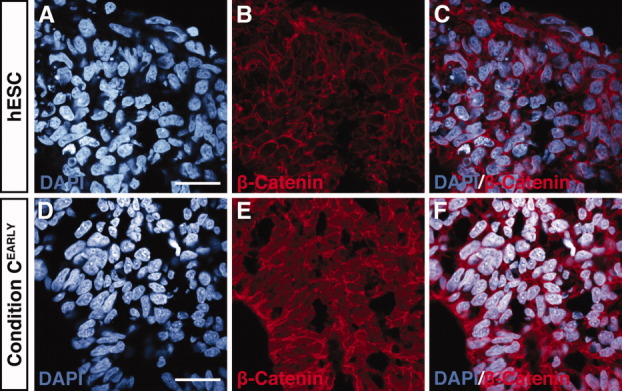
β-Catenin expression in hESC and day 4 condition C treated cultures. **(A–C):** hESC showed predominately membrane bound localization of β-Catenin. **(D–F):** CHIR- and SAG-treated cultures (condition C) at day 4, however, showed membrane bound and nuclear accumulation of β-catenin. Scale bar = 20 μm. Abbreviations: DAPI, 4′,6-diamidino-2-phenylindole; hESC, human embryonic stem cell.

### Both SHH Signaling and GSK3 Inhibition Are Required for Inducing Floor Plate Fate

Each of the conditions described above incorporated SAG throughout the entire neural induction period since it is known to be a potent inducer of ventral neural cell types. To determine whether CHIR treatment alone can induce a floor plate phenotype, hESC cultures were treated as described above with CHIR exposure for the first 4 days only, in the absence of SAG (Fig. 3, condition E). Almost no FOXA2 cells were detected after 11 days (3.3% ± 1.2% SEM; Figs. 3H, 3J′, 4A; Supporting Information Fig. 4). In contrast, several PAX6+ cells were observed, indicating that both CHIR and SAG are required for floor plate induction (Figs. 3I, 3J″). Interestingly, we also found high expression of the forebrain markers, FOXG1 and OTX2+/PAX6+, in these cultures (Supporting Information Fig. 6). This suggests that CHIR induction of hESC to a pre-neuroepithelial state retains their capacity to differentiate to forebrain neural derivatives.

**Figure 6:**
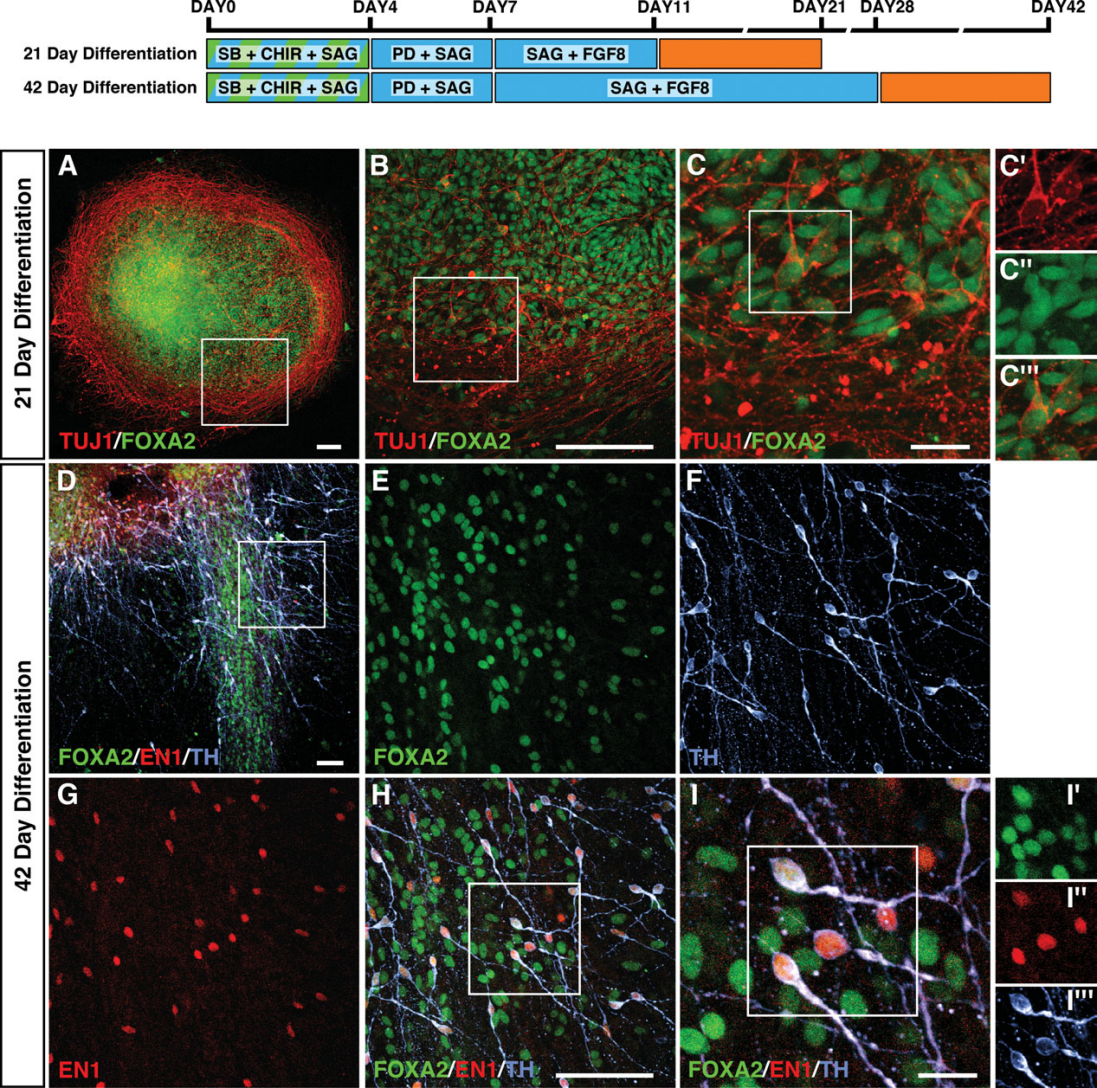
Neuronal differentiation of FOXA2+ cells yields TH/EN1/FOXA2+ neurons. Cells were cultured for 21 days **(A–C″′)** or 42 days **(D–I″′)** in vitro, whereby the final 10 and 14 days, respectively, cells were plated on laminin substrate. (A–C″′): In the 21-day neuronal differentiation conditions, cultures were examined by immunofluorescence for TUJ1+/FOXA2+ cells. (D–I″′): In the day 42 neuronal differentiation conditions, cultures were examined by immunofluorescence for TH+/EN1+/FOXA2+ cells. Scale bars = A, B, D, E, F, G, H = 100 μm and C, I = 20 μm. Time line: 21- and 42-day neuronal differentiation protocol. Orange bar indicates time on laminin substrate. Abbreviations: CHIR, CHIR-99021; SB, SB431542; SAG, smoothened agonist; PD, PD0325901; TH, tyrosine hydroxylase.

To examine whether the sequence of SAG and GSK3β inhibition signaling is critical for robust FOXA2 expression, hESC cultures were again treated with CHIR and SAG for 4 days, after which both compounds were removed (Fig. 3, condition F). These conditions resulted in significantly more numbers of FOXA2+ cells than treatment E (83.5% ± 5.9% SEM; *p* < .0001; Figs. 3N, 4A). The reverse sequence of SAG treatment was also examined, whereby hESC cultures were treated with CHIR and no SAG for the first 4 days and then removed, but SAG was then added at day 4 (Fig. 3P–3R, condition G) or at day 11 (Fig. 3S–3U, condition H), and cultures were maintained for an additional 7 days. High FOXA2+ expression (85.7% ±5.7 SEM; Fig. 4A) was only found in the former, condition G (Figs. 3Q, 4A; Supporting Information Fig. 4). In contrast, when SAG treatment was performed at later stages of neural induction (condition H), it resulted in no detectable FOXA2+ expression, however, ventralization to the forebrain basal plate domain NKX2.1 was observed (Fig. 3S–3U). This suggests that, both GSK3β inhibition and SHH signaling are required at the earliest stages of neural induction for efficient floor plate specification of hESC, and that GSK3β inhibition needs to occur first. These data are consistent with the findings described above, when CHIR was added after 4 days of SAG treatment (condition B).

### Pathways Involved in CHIR- and SAG-Mediated Induction to Floor Plate

GSK3β is involved in multiple pathways that include Shh and canonical Wnt signaling. To determine if CHIR treatment activates canonical Wnts in our induction system, subcellular localization expression of β-catenin was investigated. Nuclear staining of β-catenin was observed at day 4 in CHIR- and SAG-treated cultures (Fig. 5D–5F). These data suggest that the canonical Wnt pathway may be involved in directing hESC to a pre-neuroepithelial state.

To determine the mechanism of floor plate induction by CHIR and SAG treatments, Q-PCR analyses of FOXA2, SHH, and GLI transcription factors were performed at days 4 and 11 of differentiation. There was no significant increase in FOXA2 or SHH expression in hESC cultures after 4 days of treatment with CHIR and SAG, but when CHIR is removed at day 4, FOXA2 and SHH expression is then significantly upregulated (Fig. 4B, 4C). Interestingly, GLI1 transcript levels were significantly lower relative to hESC with CHIR treatment at day 4 (day 4 condition E; Fig. 4D). However, GLI1 was significantly increased in the CHIR/SAG-treated cultures compared to CHIR-only treated day 4 cultures (*p* = .0265; day 4 conditions E and D, respectively; Fig. 4D). In contrast, no significant changes in GLI2 and GLI3 levels were observed at the day 4 time point with CHIR only or CHIR/SAG treatments. By day 11, FOXA2 expression was upregulated with some persistent GLI1 expression in condition D cultures. This is contrasted with no significant increase in FOXA2 expression at day 11 in condition E cultures relative to hESC (Fig. 4B). Upregulated expression of GLI2 was only found in condition E at day 11, which is consistent with upregulated expression of PAX6 in these cultures (Figs. 3I, 3J, 4E). GLI3 expression was also upregulated in condition E at day 11 (*p* = .0249; Fig. 4F). Taken together, these results show that CHIR/SAG treatment of hESC prevents the onset of PAX6 expression and also prevents upregulation of GLI2/3. This allows GLI1 expression to be induced by SHH signaling shifting the balance toward a GLI activation state. GLI1 then directly upregulates FOXA2 expression to specify progenitors to a floor plate fate.

Our previous studies in hESC showed that GLI1 is a direct activator of FOXA2 expression. Thus, it may be that CHIR/SAG treatment directly induces GLI1 expression, which then upregulates FOXA2 expression in the absence of continued SHH treatment.

### Differentiation of FOXA2+ Floor Plate Cells Results in Mesencephalic Dopaminergic Neurons

A special feature of floor plate cells in the midbrain is their neurogenic ability to directly generate mesencephalic dopaminergic neurons [2, 33]. We therefore investigated whether hESC-derived FOXA2+ cells had the capacity to give rise to mesencephalic dopaminergic neurons. hESC differentiation cultures treated by condition D were further differentiated with the addition of the specific MEK1/2 inhibitor PD0325901 from days 4 to 7, which has recently been demonstrated to induce caudalization of ESCs in order to specify a midbrain identity [34]. These cultures were then separated into 21- and 42-day neuronal differentiation groups (Fig. 6). For both groups, culturing the neurospheres onto laminin substrates at day 11 and 28, respectively, induced terminal differentiation of neural progenitors. In the 21-day differentiation group, a high proportion of FOXA2+ cells differentiated into TUJ1+ neurons (Fig. 6A–6C″′), and few tyrosine hydroxylase (TH) positive neurons were observed (data not shown). For the 42-day differentiation group, robust expression of TH+ neurons were observed, and a high proportion of these colocalized expression with FOXA2 and the midbrain marker Engrailed 1/2, indicative of mesencephalic dopaminergic identity (Fig. 6D–6I″′). In conclusion, hESC can be efficiently patterned into a midbrain floor plate progenitor state, with the capacity to subsequently differentiate into mesencephalic dopaminergic neurons, through manipulation of the SHH pathway by CHIR/SAG treatment at specific time points during neural induction.

## DISCUSSION

Although the floor plate is located within the neural tube, some studies in chick and mouse have suggested that floor plate cells are specified differently to ventral neuroepithelial cells during development. The generation of the floor plate itself requires high levels of Shh, however, the timing and duration of Shh signaling is critical [9]. Our data provide evidence to support this hypothesis within the human system. Most significantly, we identified that by blocking GSK3β at the earliest stages of neural induction maintains the cells in a pre-neuroepithelial state. Exposure to SHH signaling of these pre-neuroepithelial cells allows their efficient commitment toward a floor plate fate and prevents their differentiation to PAX6+ neuroepithelium.

The intrinsic determinants of floor plate versus neuroepithelial fate include the expression of GLI transcription factors. GLI1 is an activator and a potent inducer of floor plate specification. Indeed, forced expression of GLI1 can convert neuroepithelial cells to a floor plate lineage [22]. Our data suggest that activation of GLI1 signaling via SHH can only occur in the absence of PAX6 expression, raising the possibility that GLI1 and PAX6 may act in a mutually or unidirectional repressive manner. In support of this, studies by Lek et al. [35] show that ectopic Pax6 expression within the ventral neural tube suppresses Shh signaling and floor plate specification and upregulates the Gli3 transcription factor, thereby shifting the balance of the Gli transcripts toward a repressive state. This study proposed the concept of “cellular memory,” whereby specifications of floor plate and ventral neural progenitors were dependent on the timing of SHH exposure rather than the morphogen gradient established by the dosage alone. We propose a similar mechanism within hESC whereby early cellular priming is required to establish a responsive pre-neuroepithelial state where the overriding effect of SHH signaling is ventralization through efficient activation of the GLI1 pathway in the absence of co-repressive activity through GLI2/3. This shift attains cellular memory whereby the expression of FOXA2 and commitment to a floor plate fate occurs, even in the absence of continued exogenous SHH signaling. These events mimic those in development, whereby the continued exposure of SHH is not required once the floor plate has been fated [9].

CHIR is a potent and specific inhibitor of GSK3β, which is involved in multiple signaling pathways including Wnt. Indeed, we found activation of canonical Wnt pathway at the day 4 stage of CHIR treatment, suggesting that Wnt signaling may be involved in mediating hESC to a pre-neuroepithelial state. Fasano et al. [10] showed that inhibition of the Wnt signaling via Dkk1 treatment helped induce hESC commitment toward anterior neuroectoderm and inhibition of Dkk1 resulted in an enhancement of floor plate specification. Recently others reported that CHIR is capable of inducing a caudalizing effect on hESC-derived neural progenitors into a midbrain identity, and it was suggested that this was mediated through its involvement in activating the WNT pathway [36, 37]. This mechanism is feasible given that Wnt proteins play a role in establishing the midbrain/hindbrain boundary during development [38]. Interestingly, however, our data show upregulation of the forebrain markers, NKX2.1, FOXG1, and OTX2/PAX6, in conditions H and E whereby SAG treatment was absent or delayed in the CHIR-treated hESC cultures thereby allowing upregulation of PAX6 expression. In these conditions, early exposure of CHIR only was not sufficient to induce a caudalizing effect. Overall, it is very possible that GSK3β inhibition has a dual temporal and spatial function in promoting floor plate specification and caudalization, respectively.

Although activation of the Wnt pathway may play a role in floor plate specification, we provide evidence to suggest that regulation of GLI transcription factors are also involved. Our previous studies showed that GLI1 directly induces floor plate fate in hESC [22]. Furthermore, our data in this study show that GLI2/3 transcript levels remained low with CHIR/SAG treatment, whereas GLI1 was significantly increased, suggesting that CHIR may modulate GLI signaling at the transcriptional level. Modulation of Gli transcript levels by CHIR treatment may be mediated by its destabilization of SUFU [31, 39]. Knockout of Sufu has been shown to result in the decrease in Gli2/3 protein levels and an expansion of the floor plate [28, 29]. CHIR treatment may alter the balance of GLI transcripts toward a GLI activator state when combined with SHH activation, thereby inducing FOXA2 expression and floor plate specification.

An alternative or additional mechanism mediated by CHIR may through its ability to promote self-renewal thereby extending the temporal window for cells to remain in a pre-neuroepithelial state. Other studies have shown that GSK3β inhibition promotes self-renewal of neuroepithelial cells through its regulation of the cell cycle by interacting with C-Myc and N-Myc [40, 41]. Indeed, early and continued CHIR treatment (condition C) kept the pre-neuroepithelial progenitors in a SOX2+/PAX6− state and prevented their further differentiation, perhaps mediated by the regulation of Myc family members. Furthermore, exposure of CHIR to PAX6+ progenitors (condition B) resulted in persistent PAX6 expression and suppression of NKX2.1. Given this potential function of CHIR in self-renewal, it is understandable that the timing of CHIR treatment is essential for the outcome of cellular fate.

Along the rostral-caudal axis, the floor plate generates heterogeneous populations of neural and non-neural progenitors [42]. Within the midbrain region, floor plate cells are neurogenic and give rise to mesencephalic dopaminergic neurons [2, 33]. We demonstrated that through the use of caudalizing factors, specifically a MEK1/2 inhibitor PD0325901, we could efficiently pattern the floor plate progenitors to a midbrain identity and induce neuronal commitment, which gave rise to high proportions of mesencephalic dopaminergic neurons. Recently, it was shown that blocking MEK1/2 signaling upon neural induction induces caudalization toward a midbrain identity [34]. Taken together, this methodology may be used for generating mesencephalic dopaminergic neural progenitors for drug discovery and establishing cell replacement-based approaches for treating Parkinson's Disease.

## CONCLUSION

**Figure 7:**
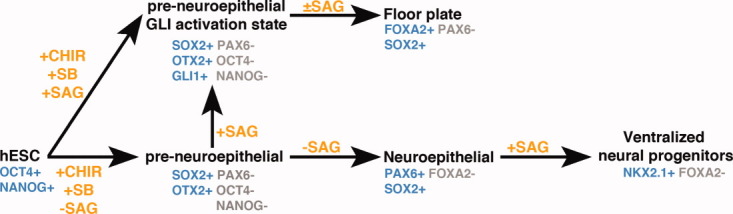
Model of floor plate and neuroepithelial specification from hESC.combined inhibition of GSK3β and activin/nodal pathways by treatment with small molecules, CHIR and SB431542, respectively, results in a pre-neuroepithelial OCT4−/NANOG−/SOX2+/OTX2+/PAX6− cellular state. Exposure to potent SHH agonist, SAG, prior to or during this period, induces expression of GLI1 to promote specification towards floor plate. In contrast, no or reduced SHH signaling results in specification towards PAX6+ neuroepithelial cells. Potent SHH signaling at the PAX6+ neuroepithelial stage results in ventralized neural progenitors, such as NKX2.1+ cells. Abbreviations: CHIR, CHIR-99021; hESC, human embryonic stem cells; SAG, smoothened agonist; SB, SB431542.

In summary, we demonstrate that development of the floor plate within the human system occurs in the same manner as what has been reported in other species (Fig. 7). The floor plate is established within a restricted developmental temporal window that exists before neuroepithelial commitment and is dependent on SHH signaling. Within human development, the onset of PAX6 expression confers a loss of floor plate competence and allows for the formation of the neuroepithelium. This temporal change is fundamentally important in that it prevents the overlying neural tube from adopting a floor plate fate since the floor plate itself secretes SHH at high concentrations. SHH secreted from the floor plate continues to pattern the ventral regions of the neural tube and demonstrates how similar SHH levels that once gave rise to floor plate can now specify basal plate identity (Fig. 7). These results also highlight how a temporal shift can confer multiple fate choice from a single morphogen concentration. Furthermore, we demonstrate that pre-neuroepithelial cells undergo a state of cellular memory in response to SHH signaling, which leads to the onset of FOXA2 conferring their commitment to a floor plate fate in the absence of continued exogenous SHH signaling. Overall, this study illustrates that responsiveness of human cells to SHH signaling to induce floor plate versus neuroepithelial fate is highly context dependent and directly linked to temporal aspects of development. These findings are significant for understanding how the floor plate develops during human embryogenesis.

## Supplementary material

Supplementary Figure 1: NKX2.1 quantification of Condition A at day 11 by FACS. (A-B) The average proportion of NKX2.1 positive cells in Condition A was 75.18% ± 7.9 SEM (n=3). FACS analysis plots of NKX2.1 were set against the side scatter and gates set according to hESCs expression levels. (C-F) Low and high power image of NKX2.1 staining of Condition A at day 11. Scale bars: C-D = 500μm and E-F =100μm.

Supplementary Figure 2: QPCR analysis of GLI1, GLI2 and GLI3 in Condition A at day 4. (A-C) Analysis of levels were compared to hESCs and the fold change levels for hESC set at 1. GLI1, GLI2 and GLI3 all showed significantly higher level in Condition A at day 4 (P < 0.05). Star indicates significance (P < 0.05).

Supplementary Figure 3: FACS analysis of Condition C Early (Day 4) for SOX2 and OCT4. (A-B) FACS analysis was performed and gates set according to hESCs for SOX2 and OCT4 expression. The average proportions of SOX2 and OCT4 positive cells for Condition C Early were 89.17% ± 2.7 SEM and 0.91% ± 0.12 SEM (n=3), respectively.

Supplementary Figure 4: FACS analysis of Conditions D-G for FOXA2. FACS analysis was performed and gates set according to hESCs. FACS analysis of Conditions D, F and G all yielded high percentages of FOXA2 positive cells, 86% (±2.8 SEM), 83.5% (±5.9 SEM) and 85.7% (±5.7 SEM) respectively. Condition E resulted in few (3.3%; ±1.2 SEM) FOXA2 positive cells, significantly lower than the other conditions (P < 0.0001). Sample size is n=3 for each condition.

Supplementary Figure 5: QPCR analysis for mesoderm and endoderm markers in Condition D and E at day11. (A-B) BRACHYURY and SOX17 expression levels were analysed in Conditions D and E and compared to hESC with the fold change levels for hESC set at 1. No significant differences in expression levels were found.

Supplementary Figure 6: Forebrain marker expression in Condition E at day 11. (AD) FOXG1 positive cells were detected throughout Condition E at day 11 and none were detected in Condition D. (E-H) OTX2+/PAX6+ cells were detected in Condition E at day 11. A-D Scale bar: 50μm

## References

[1] Tanabe Y, Jessell TM (1996). Diversity and pattern in the developing spinal cord. Science.

[2] Bonilla S, Hall AC, Pinto L (2008). Identification of midbrain floor plate radial glia-like cells as dopaminergic progenitors. Glia.

[3] Charrier JB, Lapointe F, Le Douarin NM (2002). Dual origin of the floor plate in the avian embryo. Development.

[4] Teillet MA, Lapointe F, Le Douarin NM (1998). The relationships between notochord and floor plate in vertebrate development revisited. Proc Natl Acad Sci USA.

[5] Dessaud E, McMahon AP, Briscoe J (2008). Pattern formation in the vertebrate neural tube: A sonic hedgehog morphogen-regulated transcriptional network. Development.

[6] Patten I, Placzek M (2002). Opponent activities of Shh and BMP signaling during floor plate induction in vivo. Curr Biol.

[7] Hynes M, Porter JA, Chiang C (1995). Induction of midbrain dopaminergic neurons by Sonic hedgehog. Neuron.

[8] Chiang C, Litingtung Y, Lee E (1996). Cyclopia and defective axial patterning in mice lacking Sonic hedgehog gene function. Nature.

[9] Ribes V, Balaskas N, Sasai N (2010). Distinct Sonic Hedgehog signaling dynamics specify floor plate and ventral neuronal progenitors in the vertebrate neural tube. Genes Dev.

[10] Fasano CA, Chambers SM, Lee G (2010). Efficient derivation of functional floor plate tissue from human embryonic stem cells. Cell Stem Cell.

[11] Patten I, Kulesa P, Shen MM (2003). Distinct modes of floor plate induction in the chick embryo. Development.

[12] Zhang X, Huang CT, Chen J (2010). Pax6 is a human neuroectoderm cell fate determinant. Cell Stem Cell.

[13] Pera MF, Andrade J, Houssami S (2004). Regulation of human embryonic stem cell differentiation by BMP-2 and its antagonist noggin. J Cell Sci.

[14] Davidson KC, Jamshidi P, Daly R (2007). Wnt3a regulates survival, expansion, and maintenance of neural progenitors derived from human embryonic stem cells. Mol Cell Neurosci.

[15] Aubert J, Stavridis MP, Tweedie S (2003). Screening for mammalian neural genes via fluorescence-activated cell sorter purification of neural precursors from Sox1-gfp knock-in mice. Proc Natl Acad Sci USA.

[16] Suter DM, Tirefort D, Julien S (2009). A Sox1 to Pax6 switch drives neuroectoderm to radial glia progression during differentiation of mouse embryonic stem cells. Stem Cells.

[17] Reubinoff BE, Pera MF, Fong CY (2000). Embryonic stem cell lines from human blastocysts: Somatic differentiation in vitro. Nat Biotechnol.

[18] Conley BJ, Denham M, Gulluyan L (2005). Mouse embryonic stem cell derivation, and mouse and human embryonic stem cell culture and differentiation as embryoid bodies. Curr Protoc Cell Biol.

[19] Denham M, Dottori M (2011). Neural differentiation of induced pluripotent stem cells. Methods Mol Biol.

[20] Pfaffl MW (2001). A new mathematical model for relative quantification in real-time RT-PCR. Nucleic Acids Res.

[21] Chambers SM, Fasano CA, Papapetrou EP (2009). Highly efficient neural conversion of human ES and iPS cells by dual inhibition of SMAD signaling. Nat Biotechnol.

[22] Denham M, Thompson LH, Leung J (2010). Gli1 is an inducing factor in generating floor plate progenitor cells from human embryonic stem cells. Stem Cells.

[23] Li XJ, Zhang X, Johnson MA (2009). Coordination of sonic hedgehog and Wnt signaling determines ventral and dorsal telencephalic neuron types from human embryonic stem cells. Development.

[24] Chen MH, Li YJ, Kawakami T (2004). Palmitoylation is required for the production of a soluble multimeric Hedgehog protein complex and long-range signaling in vertebrates. Genes Dev.

[25] Burke R, Nellen D, Bellotto M (1999). Dispatched, a novel sterol-sensing domain protein dedicated to the release of cholesterol-modified hedgehog from signaling cells. Cell.

[26] Chen JK, Taipale J, Young KE (2002). Small molecule modulation of Smoothened activity. Proc Natl Acad Sci USA.

[27] Svard J, Heby-Henricson K, Persson-Lek M (2006). Genetic elimination of Suppressor of fused reveals an essential repressor function in the mammalian Hedgehog signaling pathway. Dev Cell.

[28] Cooper AF, Yu KP, Brueckner M (2005). Cardiac and CNS defects in a mouse with targeted disruption of suppressor of fused. Development.

[29] Chen MH, Wilson CW, Li YJ (2009). Cilium-independent regulation of Gli protein function by Sufu in Hedgehog signaling is evolutionarily conserved. Genes Dev.

[30] Kise Y, Morinaka A, Teglund S (2009). Sufu recruits GSK3beta for efficient processing of Gli3. Biochem Biophys Res Commun.

[31] Chen Y, Yue S, Xie L (2011). Dual Phosphorylation of suppressor of fused (Sufu) by PKA and GSK3beta regulates its stability and localization in the primary cilium. J Biol Chem.

[32] Sato N, Meijer L, Skaltsounis L (2004). Maintenance of pluripotency in human and mouse embryonic stem cells through activation of Wnt signaling by a pharmacological GSK-3-specific inhibitor. Nat Med.

[33] Kittappa R, Chang WW, Awatramani RB (2007). The foxa2 gene controls the birth and spontaneous degeneration of dopamine neurons in old age. PLoS Biol.

[34] Jaeger I, Arber C, Risner-Janiczek JR (2011). Temporally controlled modulation of FGF/ERK signaling directs midbrain dopaminergic neural progenitor fate in mouse and human pluripotent stem cells. Development.

[35] Lek M, Dias JM, Marklund U (2010). A homeodomain feedback circuit underlies step-function interpretation of a Shh morphogen gradient during ventral neural patterning. Development.

[36] Kirkeby A, Grealish S, Wolf DA (2012). Generation of regionally specified neural progenitors and functional neurons from human embryonic stem cells under defined conditions. Cell Rep.

[37] Kriks S, Shim JW, Piao J (2011). Dopamine neurons derived from human ES cells efficiently engraft in animal models of Parkinson's disease. Nature.

[38] McMahon AP, Joyner AL, Bradley A (1992). The midbrain-hindbrain phenotype of Wnt-1-/Wnt-1- mice results from stepwise deletion of engrailed-expressing cells by 9.5 days postcoitum. Cell.

[39] Jia J, Amanai K, Wang G (2002). Shaggy/GSK3 antagonizes Hedgehog signalling by regulating Cubitus interruptus. Nature.

[40] Knoepfler PS, Kenney AM (2006). Neural precursor cycling at sonic speed: N-Myc pedals, GSK-3 Brakes. Cell Cycle.

[41] Kim WY, Wang X, Wu Y (2009). GSK-3 is a master regulator of neural progenitor homeostasis. Nat Neurosci.

[42] Placzek M, Briscoe J (2005). The floor plate: Multiple cells, multiple signals. Nat Rev Neurosci.

