# Supplementary figures and images for: Glycogen Synthase Kinase 3β and Activin/Nodal Inhibition in Human Embryonic Stem Cells Induces a Pre-Neuroepithelial State That Is Required for Specification to a Floor Plate Cell Lineage
Source: Stem Cells. 2012 Aug 21;30(11):2400–11. doi: 10.1002/stem.1204 (PMC3533765; doi:10.1002/stem.1204)

**A**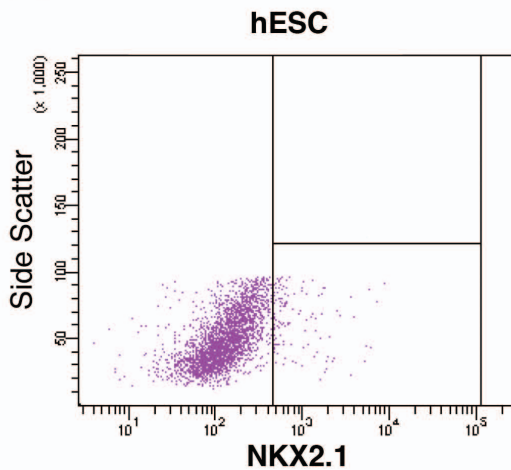**B**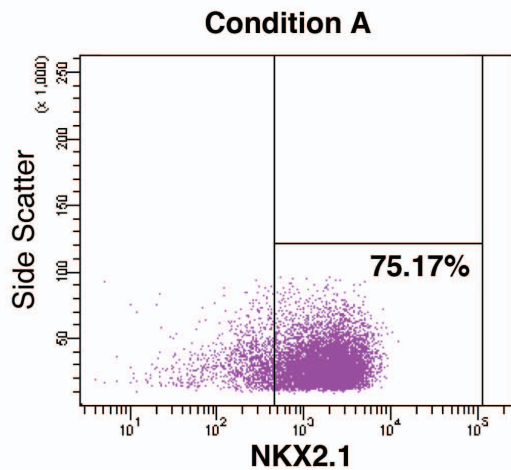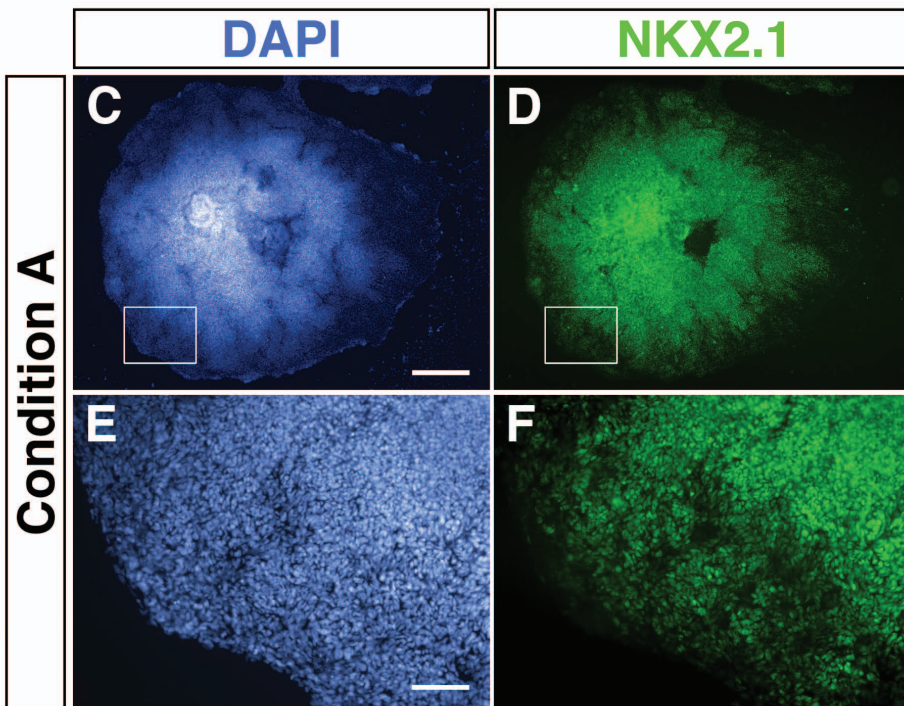

Supplement: Supplementary file 1 [file stem0030-2400-SD1.pdf]

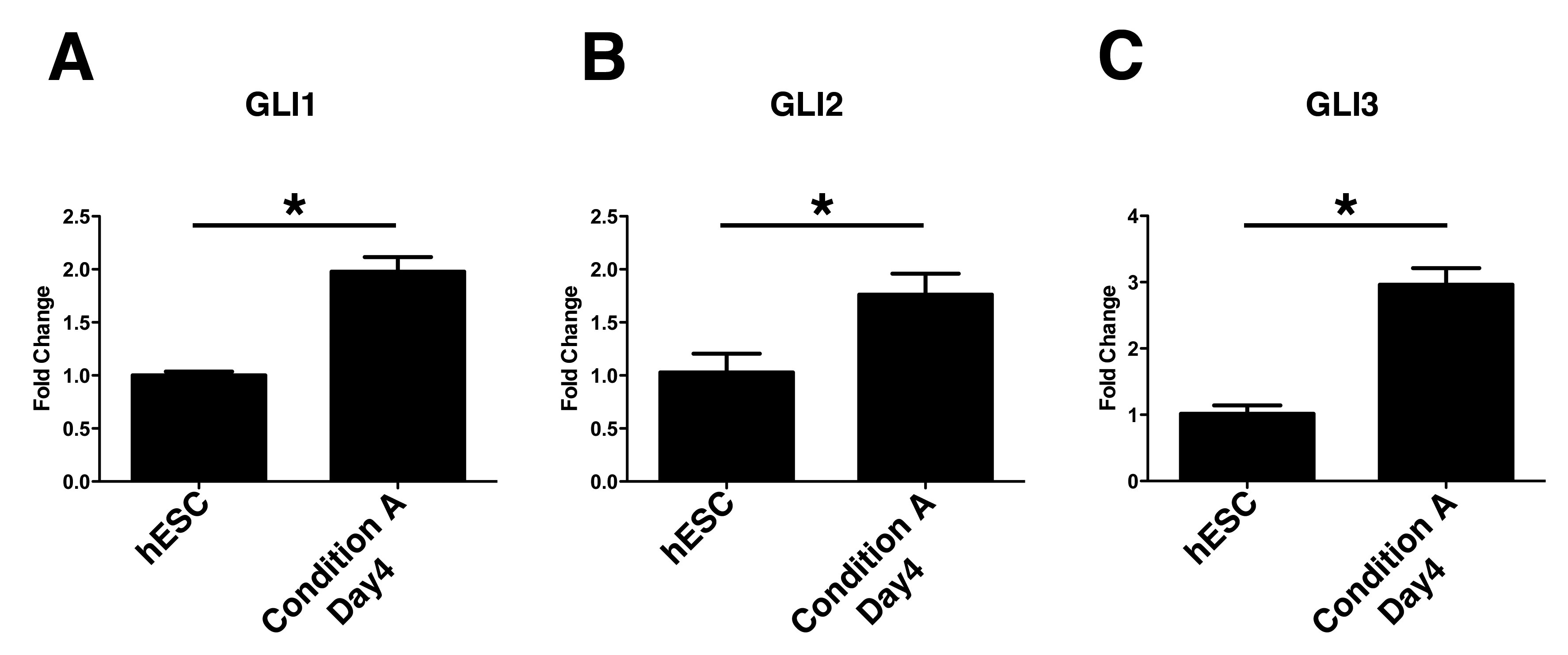

Supplement: Supplementary file 2 [file stem0030-2400-SD2.tif]

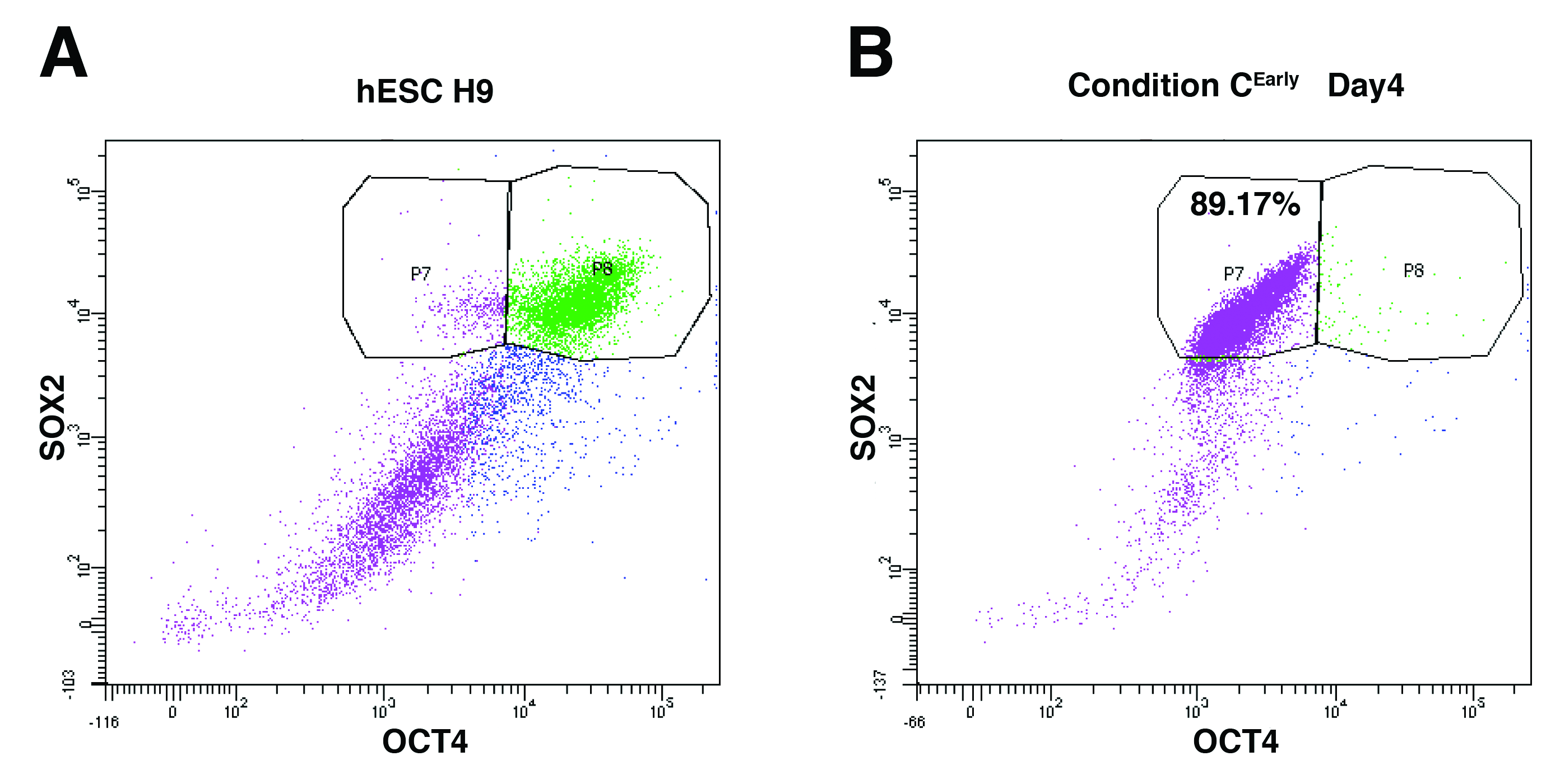

Supplement: Supplementary file 3 [file stem0030-2400-SD3.tif]

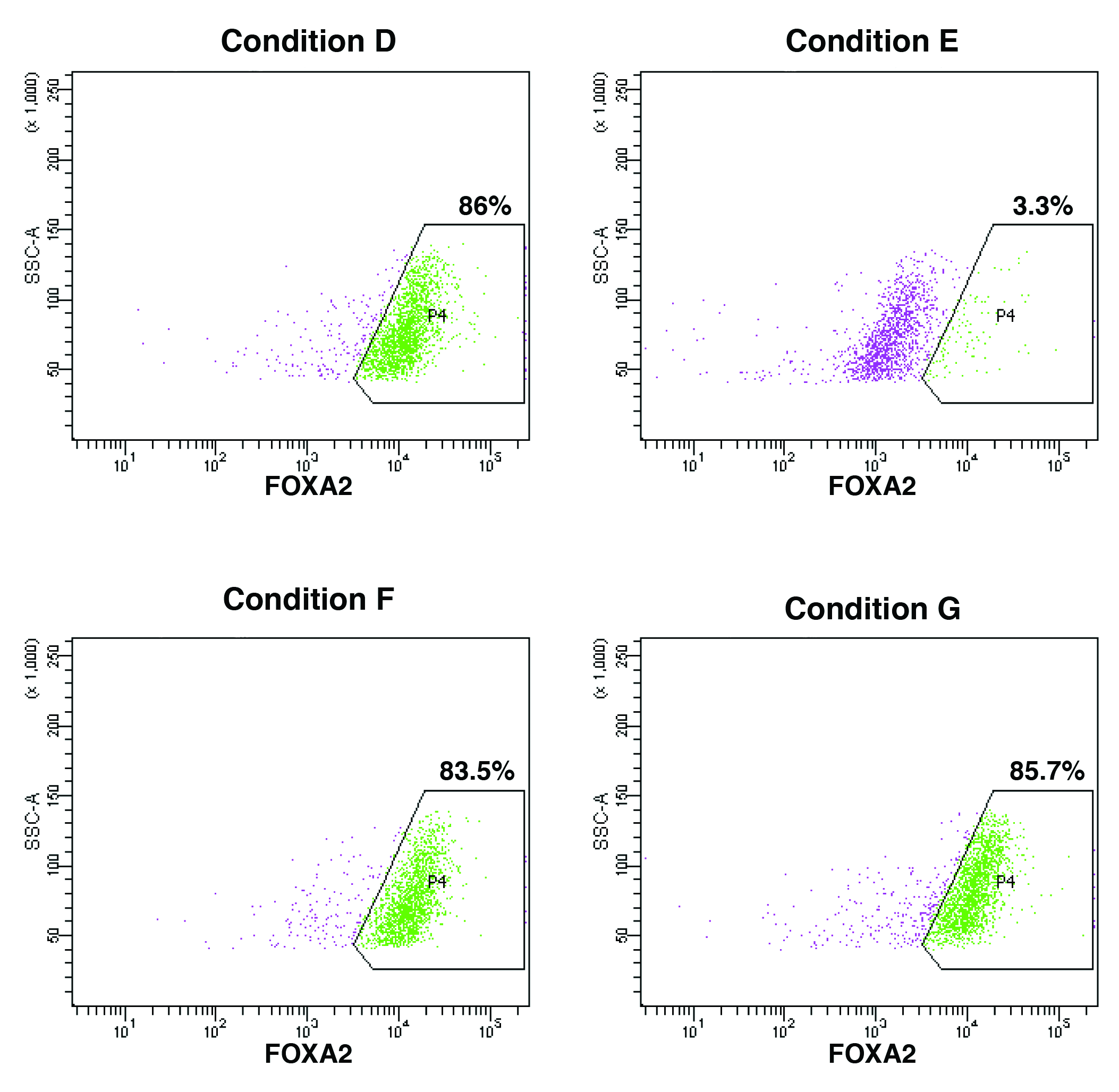

Supplement: Supplementary file 4 [file stem0030-2400-SD4.tif]

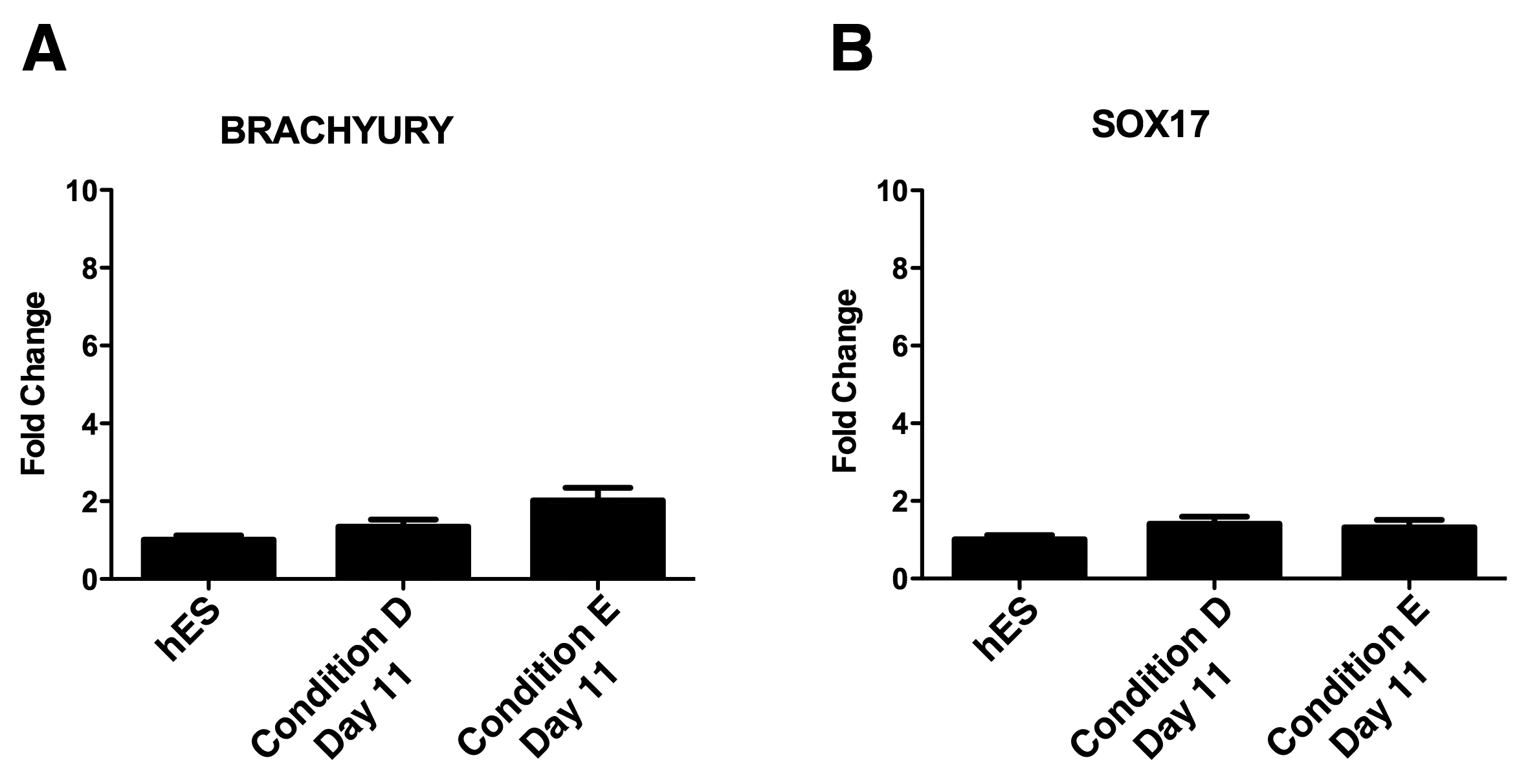

Supplement: Supplementary file 5 [file stem0030-2400-SD5.tif]

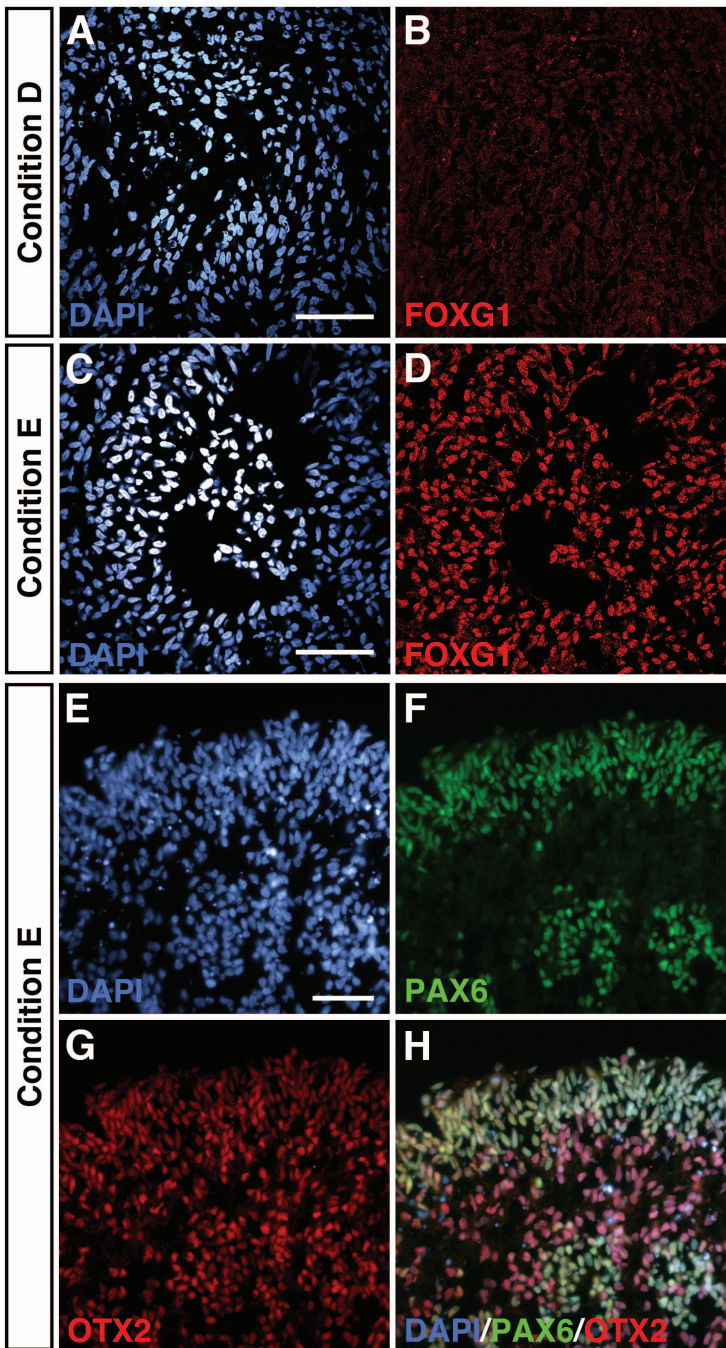

Supplement: Supplementary file 6 [file stem0030-2400-SD6.pdf]
