# Supplementary material for: Glycogen Synthase Kinase 3β and Activin/Nodal Inhibition in Human Embryonic Stem Cells Induces a Pre-Neuroepithelial State That Is Required for Specification to a Floor Plate Cell Lineage
Source: Stem Cells. 2012 Aug 21;30(11):2400–11. doi: 10.1002/stem.1204 (PMC3533765; doi:10.1002/stem.1204)
Supplement: Supplementary file 7 [file stem0030-2400-SD7.pdf]

Supplementary Methods

Differentiation Conditions:

Condition A: From days 0-11 cells were cultured on laminin. Days 0-4 medium were supplemented with SB and SAG and from days 4-11 with FGF2 and SAG.

Condition B: From days 0-11 cells were cultured on laminin. Days 0-4 medium were supplemented with SB and SAG and from days 4-11 with FGF2, SAG and CHIR.

Condition C: From days 0-4 cells were cultured on laminin and medium supplemented with SB and SAG and CHIR. On day 4 cultures were dissected into 0.5mm fragments and cultured in suspension from days 4-11, medium was supplemented with SB, SAG and CHIR.

Condition D: From days 0-4 cells were cultured on laminin and medium supplemented with SB, SAG and CHIR. On day 4 cultures were dissected into 0.5mm fragments and cultured in suspension from days 4-11, medium was supplemented with FGF2 and SAG.

Condition E: From days 0-4 cells were cultured on laminin and medium supplemented with SB and CHIR. On day 4 cultures were dissected into 0.5mm fragments and cultured in suspension from days 4-11, medium was supplemented with FGF2.

Condition F: From days 0-4 cells were cultured on laminin and medium supplemented with SB, SAG and CHIR. On day 4 cultures were dissected into 0.5mm fragments and cultured in suspension from days 4-11, medium was supplemented with FGF2.

Condition G: From days 0-4 cells were cultured on laminin and medium supplemented with SB and CHIR. On day 4 cultures were dissected into 0.5mm fragments and cultured in suspension from days 4-11, medium was supplemented with FGF2 and SAG.

Condition H: From days 0-4 cells were cultured on laminin and medium supplemented with SB and CHIR. On day 4 cultures were dissected into 0.5mm fragments and cultured in suspension from days 4-11, medium was supplemented with FGF2. From day 11-18 medium was supplemented with FGF2 and SAG.

21 Day Differentiation: From days 0-4 cells were cultured on laminin and medium supplemented with SB, SAG and CHIR. On day 4 cultures were dissected into 0.5mm fragments and cultured in suspension from days 4-7, medium was supplemented with PD0325901 and SAG. From days 7-11 medium was supplemented with SAG and FGF8. On day 11 spheres were plated on laminin and cultured in medium without the addition of factors until day 21.

42 Day Differentiation: From days 0-4 cells were cultured on laminin and medium supplemented with SB, SAG and CHIR. On day 4 cultures were dissected into 0.5mm fragments and cultured in suspension from days 4-7, medium was supplemented with PD0325901 and SAG. From days 7-28 medium was supplemented with SAG and FGF8. On day 28 spheres were plated on laminin and cultured in medium without the addition of factors until day 42.

Real-time Polymerase Chain Reaction Primers

Primers against the human genes:

FOXA2 (forward GCCCGAGGGCTACTCCTCCG and reverse TCATGTTGCCCGAGCCGCTG),
GLI1 (forward GCCAGAGGGTGCCATGAAGCC and reverse GCCCCCTGCATTGCCAGTCA),
GLI2 (forward CCCGGGAAGTGGCTCCATCCT and reverse AGCATGGTCACCTCGCTCGC),
GLI3 (forward TGCAATCAGCCCTGCCTTGAGC and reverse TGGAGGGCTGTGTCCAAAGGCT),
SHH (forward CACCACGTCTGACCGCGACC and reverse CCGAGTTCTCTGCTTTCACCGAGC),
BRACHYURY (forward CCCCGACTCGCCCAACTTCG and reverse AGGAGTTCAGCATGATCTGGCCCC),
SOX17 (forward ATGCTGGGCAAGTCGTGGAAGG and reverse GCCGCGGCCGGTACTTGTAG)
and the housekeeping gene HPRT1 (forward CTTGCTGCGCCTCCGCCT and reverse ATCACTAATCACGACGCCAGGGC) were designed using Primer3 [1].

References

1. Rozen S, Skaletsky H. Primer3 on the WWW for general users and for biologist programmers. Methods Mol Biol. 2000;132:365-386.
